# Supplementary material for: Coupling a single spin to the motion of a carbon nanotube
Source: Nat Commun. 2025 Dec 13;16:11454. doi: 10.1038/s41467-025-66331-5 (PMC12749144; doi:10.1038/s41467-025-66331-5)
Supplement: Supplementary file 1 — Supplementary Information [file 41467_2025_66331_MOESM1_ESM.pdf]

Supplementary Information for “Coupling a single spin to the motion of a carbon nanotube”

Federico Fedele,[1*] Federico Cerisola,[1,2*] Lea Bresque,[3] Florian Vigneau,[4]
Juliette Monsel,[5] Jorge Tabanera-Bravo,[6] Kushagra Aggarwal,[4] Jonathan Dexter,[4]
Sofia Sevitz,[7] Joe Dunlop,[2] Alexia Auffeves,[8,9] Juan MR Parrondo,[6]
Andras Palyi,[10,11] Janet Anders,[2,7] Natalia Ares[1†]

[1]Dept. of Engineering Science, University of Oxford, Oxford OX1 3PJ, United Kingdom.
[2]Dept. of Physics and Astronomy, University of Exeter, Exeter EX4 4QL, United Kingdom.
[3]Univ. Grenoble Alpes, CNRS, Grenoble INP, Institut Néel, 38000 Grenoble, France.
[4]Dept. of Materials, University of Oxford, Oxford OX1 3PH, United Kingdom.
[5]Dept. of Microtechnology and Nanoscience (MC2), Chalmers University of Technology, S-412 96 Göteborg, Sweden.
[6]Dept. of Structure of Matter, Thermal Physics and Electronics and GISC, Universidad Complutense de Madrid, Pl. de las Ciencias 1. 28040 Madrid, Spain.
[7]Institute of Physics and Astronomy, University of Potsdam, 14476 Potsdam, Germany.
[8]MajuLab, CNRS-UCA-SU-NUS-NTU International Joint Research Laboratory, Singapore, Singapore.
[9]Centre for Quantum Technologies, National University of Singapore, 117543 Singapore, Singapore.
[10]Department of Theoretical Physics, Institute of Physics, Budapest University of Technology and Economics, Muegyetem rkp. 3., H-1111 Budapest, Hungary.
[11]HUN-REN-BME-BCE Quantum Technology Research Group, Budapest University of Technology and Economics, Műegyetem rkp. 3., H-1111 Budapest, Hungary

*These authors contributed equally to this work.
†Corresponding author. Email: natalia.ares@eng.ox.ac.uk

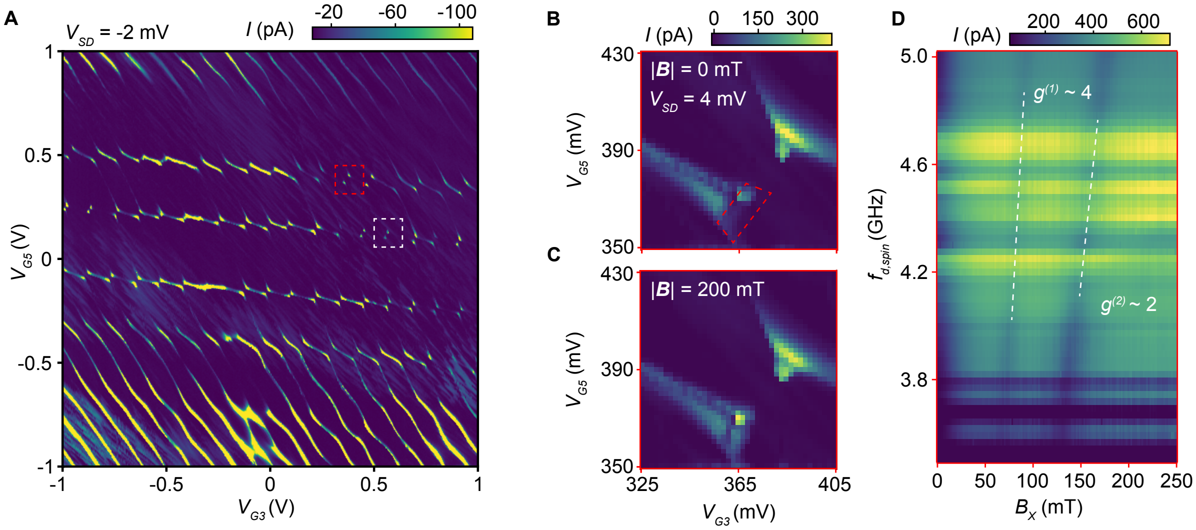

Supplementary Fig. 1: **Pauli spin blockade and EDSR spectrum for a different charge transition.** (**A**) Charge stability diagram of the double quantum dot measured as a function of V_{G3} and V_{G5} with $V_{SD} = -2$ mV. As a function of V_{G5} we can tune the system into a double quantum dot. The white dashed box indicates the pair of bias triangles used in the main text (see Fig. 1**B**-**C**), which shows Pauli blockade at negative source-drain bias. Similarly, in panels **B** and **C** we show the current measured with a positive bias $V_{SD} = +4$ mV, probed in the vicinity of the bias triangles enclosed by the red dashed box, for different magnetic fields magnitudes $|\boldsymbol{B}| = 0$ mT and $|\boldsymbol{B}| = 200$ mT respectively. At zero magnetic field, the current at the base of the lower left bias triangle is suppressed by Pauli blockade, see the red dashed box in panel **B**. (**D**) EDSR spectrum as a function of the drive frequency $f_{\mathrm{d,spin}}$ and applied field B_{X}. Like the data presented in the main text, we identify two EDSR resonances as regions of suppressed current with g-factors respectively $g^{(1)} \approx 4$ and $g^{(2)} \approx 2$ (see white dashed lines).

S1 Forward bias EDSR and Qubit location

To confirm the EDSR signal, we measured it at a different charge transition with opposite source-drain bias. In Supplementary Fig. 1**A** we present a stability diagram obtained by measuring the current I as a function of gate voltages V_{G5} and V_{G3}, which exhibit both single and double quantum dot regimes. Within the double dot regime, we selected two pairs of bias triangles, highlighted by the white and red dashed boxes, that exhibited signatures of Pauli spin blockade. As shown in the main text, the pair of bias triangles within the white box exhibits Pauli-blockade when transport is measured using a negative source-drain bias, while the pair of triangles highlighted by the dashed red box exhibits signatures of Pauli spin-blockade on the lower triangle when a positive bias is used. Supplementary Fig. 1**B-C** shows Pauli blockade at this particular pair of bias triangles measured with a source-drain bias of $V_{SD} = +4$ mV. Similar to Fig. 1**B** of the main text, at $B_{\mathrm{X}} = 0$ mT, the current at the base of the lower left bias triangle is suppressed by Pauli blockade, see the red dash box in Supplementary Fig. 1**B**.

Measuring the current within this region in the presence of a continuous wave excitation $f_{\rm d,spin}$, reveals the EDSR spectrum presented in Supplementary Fig. 1**D**. Consistent with the data presented in the main text, even in forward bias the two observed EDSR resonances are evident as regions of suppressed currents with estimated g-factors of 2 and 4, respectively. We find that this observation, considering that no EDSR was detected outside either of the Pauli-blockaded regions, confirms that we can define spin qubits in our CNT device.

S2 Magneto transport spectroscopy and EDSR as a function of field orientation

Supplementary Fig. 2**A** shows magneto transport spectroscopy measured as a function of gate voltage V_{G5} and field orientation in the X-Z plane of our reference frame (see Fig. 1**A** in the main text.) Throughout this measurement, we fixed the magnetic field magnitude to 500 mT, and set V_{G3} in order to probe the region of current rectified by Pauli blockade as a function of V_{G5}. As a function of the field angle θ, we observe two regions of suppressed current with 180° periodicity at angles θ = 55° and 235°, and angles 145° and 235°. We attribute the regions of strongest (weakest) current suppression to the direction for which the field aligns parallel or anti-parallel with (perpendicular to) the main axis of the carbon nanotube. Supplementary Fig. 2**B** shows EDSR resonances measured with the field oriented along the X direction of the reference frame. Supplementary Fig. 2**C** shows EDSR resonances measured with the field oriented along the direction of strongest current suppression, which we labelled $B_{||}$ (see the white dashed line in panel **A** at θ = 55°).

S3 Power dependence of the spin mechanical coupling

In Supplementary Fig. 3, we show additional data displaying the off-resonant spin mechanical coupling as a function of the mechanical drive power $P_{\rm d,osci}$. From panels **A** to **E**, $P_{\rm d,osci}$ is stepped from -55.5 dBm to -30.5 dBm, with panels **A** and **D** corresponding respectively to the data presented in Fig. 2**A** and 2**B** of the main text. With increasing $P_{\rm d,osci}$, we observe a broadening ($\Delta B_{\rm X}$) of the EDSR resonance when $f_{\rm d,osci} \sim f_{\rm m}$. The broadening is obtained by comparing the width of the EDSR at $f_{\rm d,osci} = 261.65$ MHz, with its width at $f_{\rm d,osci} = f_{\rm m}$. Here, the width is defined as the range of field values where the current I is within a factor $1/\sqrt{2}$ larger than the minimum EDSR current (see white dashed lines). In panel **F** top we plot the estimated ($\Delta B_{\rm X}$) divided by 2, as a function of the drive power $P_{\rm d,osci}$ and the corresponding carbon nanotube displacement δz. As discussed in the main text, the broadening shows a non-linear dependence on the driving power, an effect of the nonlinear nature of the oscillator. In Supplementary Fig. 3**G** we present further evidence for the nonlinearity of the mechanical oscillator. The current traces as a function of $f_{\rm d,osci}$ are linecuts extracted from Supplementary Fig. 3**A**-**E** and highlight the hardening of the mechanical frequency $f_{\rm m}$ as a function of $P_{\rm d,osci}$. Previous studies on CNT resonators have found these signatures to be consistent with a Duffing oscillator.[2,3] Recently, Duffing oscillators in CNT resonators have been proposed as a platform to realise nanomechanical qubits.[4]

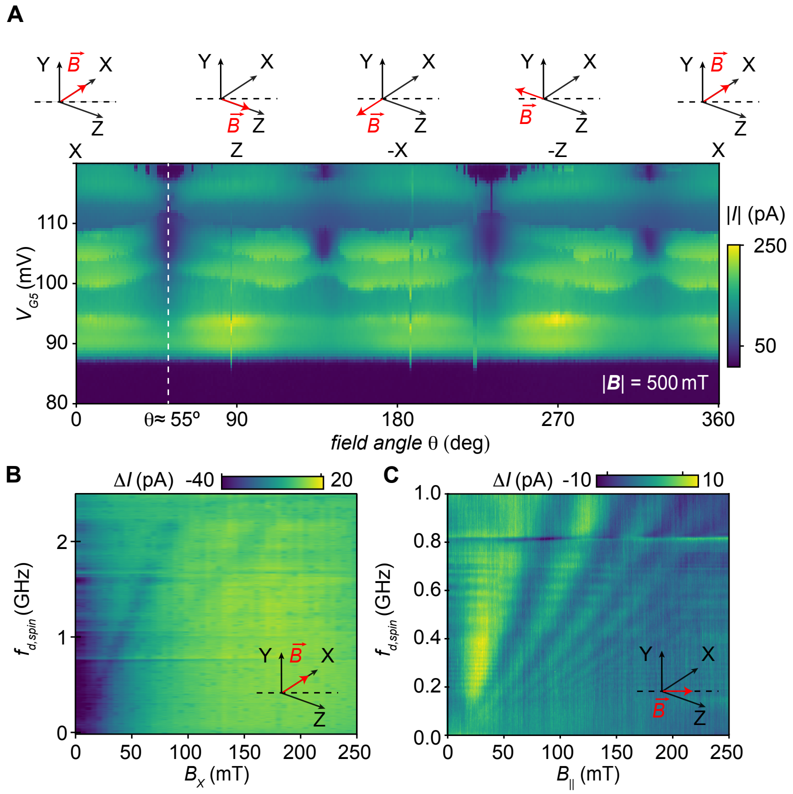

Supplementary Fig. 2: **Magneto transport angular dependence and EDSR at different field orientations.** (**A**) Current measured as a function of V_{G5} and the angle θ which determines the magnetic field orientation in the XZ plane in our frame of coordinates. The magnitude of the magnetic field was kept constant at 500 mT. Supported by geometrical considerations, we identify these regions of suppressed current with the directions for which the field aligns along the main carbon nanotube axis. The angle θ = 55°, highlighted by a white dashed line marks the orientation that we called $B_{||}$ in the rest of the manuscript. (**B**) EDSR resonances measured with the field aligned along the X-axis of our reference frame (adapted from Fig. 1**E** of the main paper). (**C**) EDSR resonances measured at the field orientation $B_{||}$.

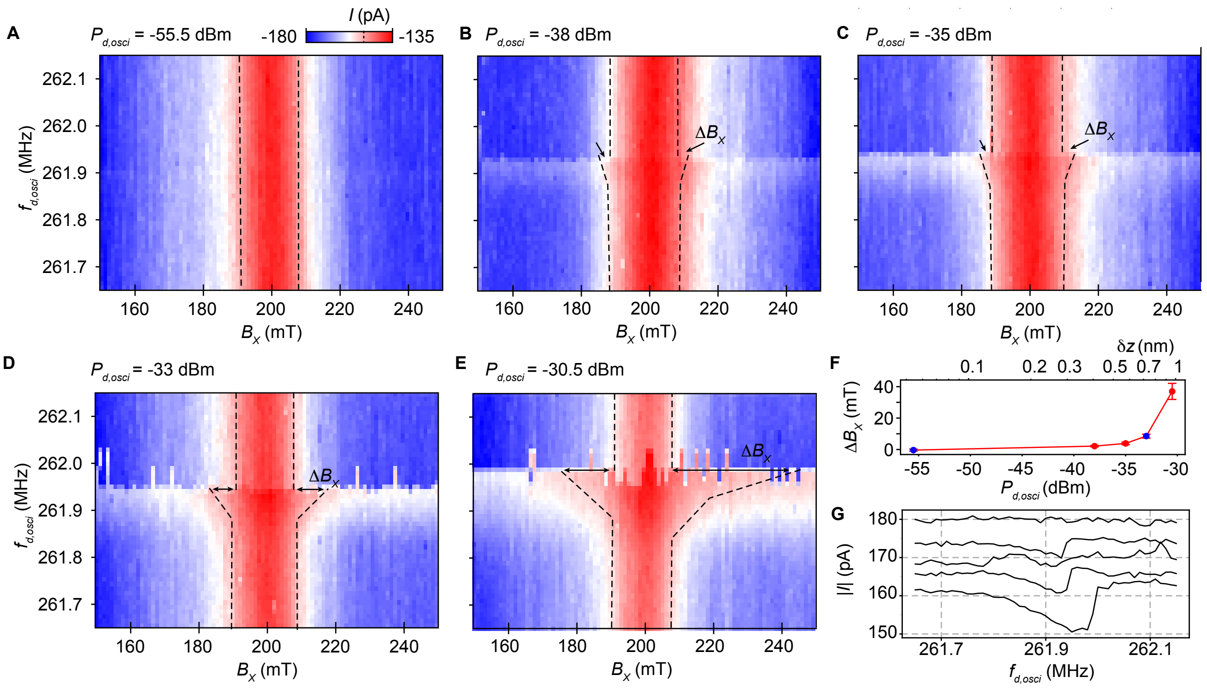

Supplementary Fig. 3: **Power dependence of the spin mechanical coupling.** (**A**)-(**E**) Non-resonant spin mechanical coupling for increasing mechanical drive power $P_{\mathrm{d,osci}}$, from -55.5 dBm to -30.5 dBm. Panel **A** and **D** are the same as Fig. 2 **A**-**B** in the main text. With increasing values of $P_{\mathrm{d,osci}}$, the EDSR resonance broadens (ΔB_{X} indicated by arrows) while approaching the mechanical resonance frequency f_{m} (see panel **F** top). Seemingly, with increasing driving power, the mechanical resonance shifts to higher frequencies due to the non-linearity of the mechanical oscillator. (**F**) Broadening of the EDSR resonance peak at $f_{\mathrm{d,osci}} \sim f_{\mathrm{m}}$ as a function of the mechanics driving power $P_{\mathrm{d,osci}}$. The blue dots indicate the estimated broadening of the data presented in the main text. On the top axis, we report the overall nanotube displacement δz estimated from $P_{\mathrm{d,osci}}$ using a standard electrostatic model with the CNT mass, physical dimensions, and capacitance to the electrostatic gates obtained from previous experiments.[1] (**G**) Linecuts extracted from **A** to **E** going from top to bottom (traces are offset for clarity), at B_{X} = 185 mT, showing the current as a function of $f_{\mathrm{d,osci}}$, for different excitation powers.

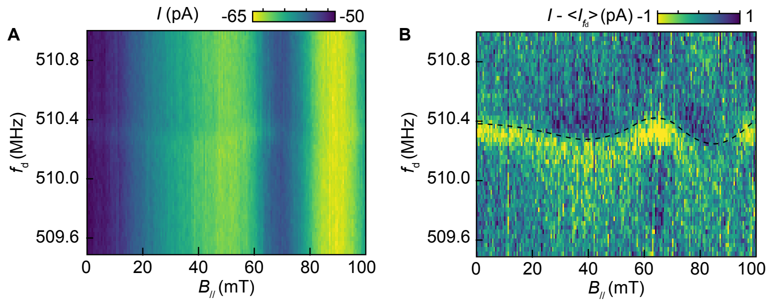

Supplementary Fig. 4: **Charge coupling to second mechanical mode:** (**A**) Measured EDSR current I, as a function of the magnetic field $B_{||}$ and a single continuous driving frequency f_d swept over a small frequency range in the vicinity of the 2nd mechanical mode (approx. 510.4 MHz). We note that, unlike Fig. 3A of the main text, here we do not observe the EDSR peak shift, characteristic of spin-mechanical coupling. (**B**) Same data as in panel **A** after subtracting the column-wise average current ($\langle I_{f_\text{d}} \rangle$) from each corresponding column (i.e. $I - \langle I_{f_\text{d}} \rangle$). The black dashed line highlights a slight hardening of the mechanical mode at the EDSR resonance, consistent with charge–mechanical coupling.

S4 Charge coupling to higher mechanical modes

Supplementary Fig. 4 presents the EDSR current measured as a function of the magnetic field strength, when the field is parallel to the CNT main axis ($B_{||}$) and a single driving frequency f_d. The driving frequency is swept in the vicinity of a mechanical resonance near 510.4 MHz, which we identify with the second mechanical mode, with a power of -33 dBm reaching gate G2. In contrast to Fig. 3**A** of the main text, we do not observe a shift in the EDSR resonance typically associated with spin–mechanical coupling. Instead, we note a weak dependence of the mechanical resonance frequency on the EDSR current. To highlight this effect, Supplementary Fig. 4**B** presents the same data as panel A, but with the column-wise average current $< I_{f_\text{d}} >$ subtracted from each column to enhance contrast. A black dashed line indicates the subtle hardening of the mechanical resonance near the EDSR current peak around $B_{||}$ = 70 mT. This behaviour is qualitatively different from the signature of spin–mechanical coupling in Fig. 3**A** and is instead consistent with conventional charge–mechanical coupling.

S5 Sweep direction effects on spin-mechanical signatures

Here we present data of the spin mechanical signatures, both resonant and off-resonant, as a function of different drive frequency sweep directions.

In Supplementary Fig. 5 **A**, we show a schematic of the device indicating the gates used to apply the two independent drive tones: one for exciting EDSR (applied to G4) and the other for driving the mechanical motion of the CNT (applied to G2). The orientation of the magnetic field relative to the CNT axis is also indicated.

Supplementary Fig. 5**B** and **C** present measurements of off-resonant spin–mechanical coupling, recorded while sweeping the mechanical drive frequency $f_{d,osci}$ in opposite directions:

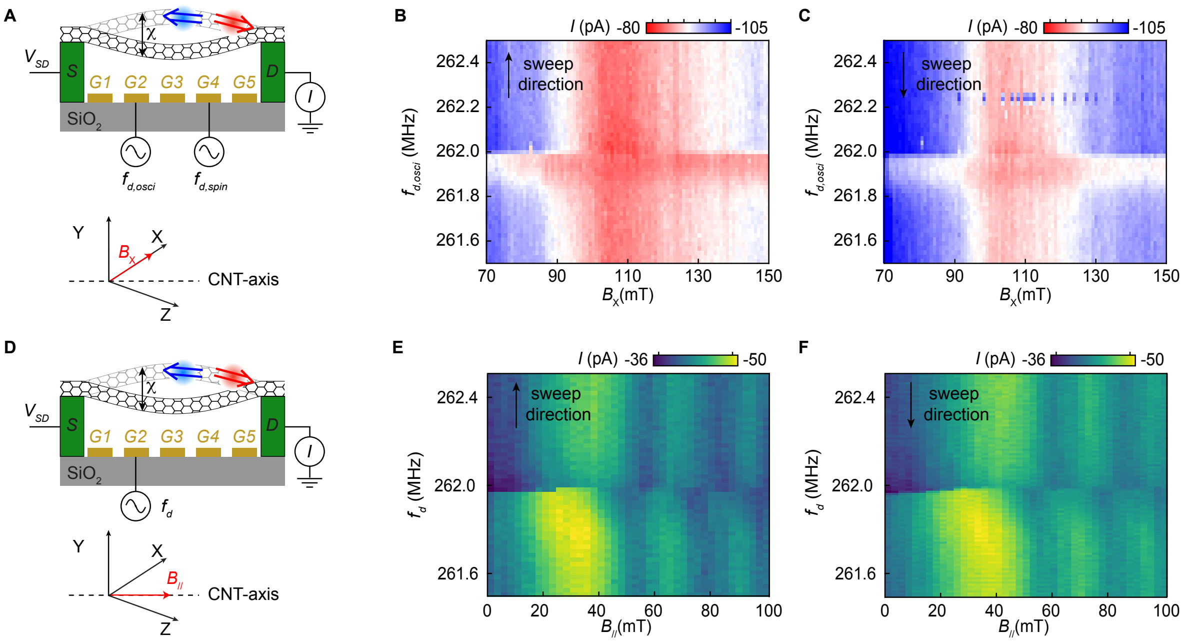

Supplementary Fig. 5: **Spin mechanical signatures for different frequency sweep directions:** (**A**) Schematic of the device and magnetic field orientation for the non resonant spin-mechanical coupling measurement scheme. Two separate continuous-wave drives $f_{\mathrm{d,osci}}$ and $f_{\mathrm{d,spin}}$ are used to separately excite the CNT mechanical motion and EDSR. (**B-C**) Measurements of the EDSR current I as a function of transverse magnetic field B_{X} and drive frequency $f_{\mathrm{d,osci}}$ recorded with an up-sweep (panel B), and a down-sweep (panel C) of the drive frequency $f_{\mathrm{d,osci}}$. (**D**) Field and device schematic for the resonant spin-mechanical coupling measurement scheme. Here, a single continuous-wave drive f_{d} excites both the mechanical motion and the EDSR current, in the vicinity of a mechanical resonance. (**E-F**) EDSR current I as a function of parallel magnetic field $B_{||}$ and drive frequency f_{d}, recorded with an upward sweep (E) and a downward sweep (F) of the drive frequency.

from low to high frequency in panel B, and from high to low in panel C. During these measurements, the EDSR drive frequency was set to 3.9 GHz, detuned from any known mechanical resonances, and resulting in an EDSR resonance around $B_{\mathsf{X}} \sim 110$ mT. The power used for the drive tones were $P_{\mathrm{d,spin}}$ = -31.25 dBm and $P_{\mathrm{d,osci}}$ = -43 dBm respectively for the spin and the mechanics. In panel C, the current switches observed near 262.2 MHz are consistent with the non-linear response of the mechanical oscillator with downward sweep-direction.

In Supplementary Fig. 5**D–F**, we present data for the resonant spin–mechanical coupling regime. Here, a single drive tone is used to simultaneously excite both the mechanical motion and the EDSR current (see panel D). Panel E shows a measurement performed with a low-to-high frequency sweep of the drive tone, while panel F (adapted from Fig. **??A** in the main text) was acquired using a high-to-low frequency sweep. Note that panel E was measured at a lower resolution (50 points between 261.5 and 262.5 MHz) compared to panel F, which was acquired with 100 points. Both measurements have been taken with a drive power of P_{d} = -33 dBm using the lock-in chopped measurement technique described in the main text.

S6 Detuning dependence of the spin mechanical coupling

In Supplementary Fig. 6**A** we show EDSR resonances measured as a function of drive power P_{G2} and applied field $B_{||}$ with a single drive frequency such that $f_{\mathrm{d,osci}} = f_{\mathrm{d,spin}} = f_d = f_{\mathrm{m}} = 261.9$ MHz. This allows us to drive the spin while simultaneously driving the mechanics and investigating the coupling as a function of the driving powers. The fixed frequency driving reveals a fan of EDSR resonances. As a function of P_d we observe a shift in the EDSR resonances towards higher values of the applied field $B_{||}$. We note that this is in contrast with the data presented in Fig. 3**A** of the main text, where the observed EDSR resonances shift towards lower values of the applied field as a function of the drive frequency.

To investigate this further, we take similar measurements at different detuning values. Supplementary Fig. 6**B** shows the charge stability with circles indicating the detuning corresponding to Supplementary Fig. 6**A** and Supplementary Fig. 6**C** to **L**. Note that the detuning of Fig. 3**A** in the main text is highlighted with a star. As a function of detuning, we observe a distinct change in the sign of the EDSR resonant shift, with panels **C**-**G** exhibiting a shift towards lower values of the applied external field, panel **H** showing no appreciable shift, and panels **I**, **L**, and **A** showing a marked shift toward higher values of the magnetic field. This change in the sign of the spin-resonance shift indicates that we could in principle tune the coupling between the spin and the mechanical motion. We attribute this tunability to the modulation of the g-tensor components with gate voltages V_{G3} and V_{G5}.

The model employed to explain the origin of the spin–mechanical coupling suggests that in the resonant case, the coupling parameter λ_2 depends on the difference of the squares of the g-factors components, $g_\perp$ and $g_{||}$. This can in principle lead to variable coupling strength owing to a modulation of the g-factor components with detuning. g-tensor modulation with gate voltages and applied magnetic field has been observed in a variety of materials including SiGe quantum dots,[5] Ge/SiGe heterostructures,[6] and CMOS silicon on insulator quantum dots devices.[7,8]

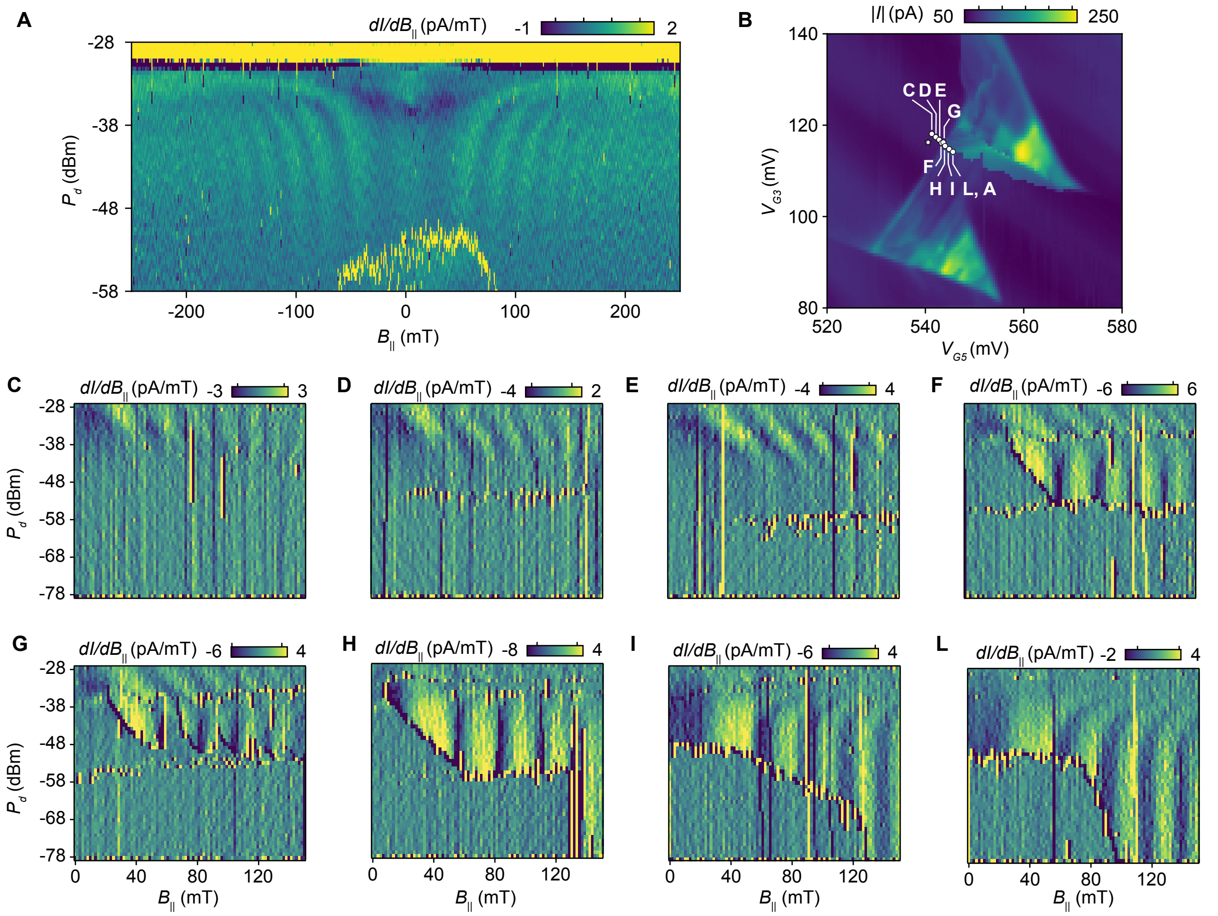

Supplementary Fig. 6: **Detuning dependence of the spin-mechanical coupling.** (**A**) here we show $dI/dB_{||}$ revealing EDSR resonances as a function of applied parallel field $B_{||}$ and drive power P_d at a fixed drive frequency $f_d = 261.9$ MHz. For increasing values of P_d the EDSR resonances shift significantly towards higher values of $B_{||}$. (**B**) Charge stability diagram of the double dot with markers indicating the detunings at which panels **A** and **C**-**L** have been measured. The star indicates Fig. 3**A** in the main text. Panels (**C**)-(**L**) show EDSR resonances measured as a function of P_d and $B_{||}$ at $f_d = 261.9$ MHz. Each panel is measured at a different detuning point highlighted by circles in panel **B**. Like in panel **A**, we observe a shift in the EDSR resonances as a function of P_d. Similar to Fig. 3**A** in the main text, in panels **C** to **G** the observed shift occurs towards lower values of $B_{||}$. In panel **H**, we do not observe any appreciable shift, while similar to panel **A**, panels **I** and **L** show the resonances shifting towards higher values of the applied field.

S7 Theoretical model

The Hamiltonian of the spin-valley qubit in the presence of a magnetic field $\mathbf{B}$ is in general given by

$$H_0 = \frac{1}{2}\mu_{\mathrm{B}}\mathbf{B}\cdot\mathbf{g}\cdot\boldsymbol{\sigma}, \tag{1}$$

where $\mathbf{g}$ is the anisotropic g-tensor.[9,10] The off-and-on resonance experiments described in the main text were performed with two different field orientations on a plane containing the CNT. As such, without loss of generality, we define a parallel ($B_{||}$) and perpendicular ($B_{\perp}$) component of the magnetic field to the CNT at rest, so that the third component of $\mathbf{B}$ is zero for all measurements. The Hamiltonian for the CNT at rest is then given by

$$H_{\mathrm{rest}} = \frac{1}{2}\mu_{\mathrm{B}}(B_{||}g_{||}\sigma_{||} + B_{\perp}g_{\perp}\sigma_{\perp}) = \frac{1}{2}\mu_{\mathrm{B}}\sqrt{B_{||}^2 g_{||}^2 + B_{\perp}^2 g_{\perp}^2}\,\sigma_3 = \frac{1}{2}E_{\mathrm{rest}}\sigma_3, \tag{2}$$

where σ_3 is the Pauli operator in the direction of the energy quantisation axis of the qubit.

If the CNT oscillates within this same plane defined by magnetic field orientations $B_{||}$ and $B_{\perp}$, then under a displacement χ of the CNT, the parallel and perpendicular field components, with respect to the CNT main axis, rotate by an angle $\tan\theta = \ell/\chi$, where ℓ is the distance of the quantum dot to the closest lead (see Fig. 1**A** of the main text). The parallel and perpendicular components of the field change according to

$$B_{||} \longrightarrow \frac{B_{||}\,\ell - B_{\perp}\,\chi}{\sqrt{\chi^2+\ell^2}}, \qquad B_{\perp} \longrightarrow \frac{B_{\perp}\,\ell + B_{||}\,\chi}{\sqrt{\chi^2+\ell^2}}. \tag{3}$$

The Hamiltonian in the new quantisation axis is then

$$H_0 = \frac{1}{2}\mu_{\mathrm{B}}\sqrt{\frac{(B_{||}\,\ell - B_{\perp}\,\chi)^2 g_{||}^2}{\chi^2+\ell^2} + \frac{(B_{\perp}\,\ell + B_{||}\,\chi)^2 g_{\perp}^2}{\chi^2+\ell^2}}\,\sigma_3. \tag{4}$$

Expanding to the second order in χ we then have

$$H_0 = \frac{1}{2}E_{\mathrm{rest}}\sigma_3 + \lambda_1\chi\sigma_3 + \lambda_2\chi^2\sigma_3, \tag{5}$$

where

$$\lambda_1 = \mu_{\mathrm{B}}^2\frac{B_{\perp}B_{||}\left(g_{\perp}^2 - g_{||}^2\right)}{2\ell E_{\mathrm{rest}}}, \tag{6}$$

$$\lambda_2 = \mu_{\mathrm{B}}^4\frac{\left(B_{||}^4 g_{||}^2 - B_{\perp}^4 g_{\perp}^2\right)\left(g_{\perp}^2 - g_{||}^2\right)}{4\ell^2 E_{\mathrm{rest}}^3}. \tag{7}$$

We will here consider the mechanics as a classical system and hence χ will be a classical

displacement satisfying the equations of motion of a Duffing oscillator,[2–4] that is

$$\ddot{\chi} + \nu\dot{\chi} + \omega_m^2\chi + \beta\chi^3 = \frac{F_0}{m}\cos(\omega_d t), \tag{8}$$

where ν is the damping coefficient of the mechanics, β the Duffing coefficient, F_0 the driving force, and m the mass of the CNT. To deal with the Duffing equation analytically or numerically it is very convenient to rewrite the equations of motion in dimensionless quantities

$$\bar{t} = t\omega_m, \quad \bar{\omega} = \frac{\omega}{\omega_m}, \quad \bar{\chi} = \frac{m\omega_m^2}{F_0}\chi, \quad \bar{\nu} = \frac{\nu}{\omega_m}, \quad \bar{\beta} = \frac{\beta F_0^3}{m^3\omega_m^8}. \tag{9}$$

so that

$$\ddot{\bar{\chi}} + \bar{\nu}\dot{\bar{\chi}} + \bar{\chi} + \bar{\beta}\bar{\chi}^3 = \cos(\bar{\omega}_d\bar{t}). \tag{10}$$

In what follows we will focus on the case of very weak non-linearity, $\bar{\beta} \ll 1$. In this regime, the response of the oscillator is approximately harmonic, with an amplitude given by the frequency-dependent Duffing response. The values of $\bar{\beta}$ extracted from the experimental data and shown in the next section are consistent with this assumption.

The qubit is further driven by the EDSR pulse, which induces Rabi oscillations. Assuming the condition for the rotating wave approximation (RWA) we then have the effective qubit Hamiltonian

$$H = H_0 + \Omega\sigma_1, \tag{11}$$

where Ω is the Rabi frequency.

Finally, the qubit in the experiment is subject to different sources of noise that induce strong decoherence. To account for this effect, we use a standard pure-dephasing master equation

$$\dot{\rho} = -\frac{i}{\hbar}[H, \rho] + \Gamma\left(\sigma_3\rho\sigma_3 - \rho\right), \tag{12}$$

where Γ determines the decoherence time. For the case where decoherence is dominated by pure dephasing,[11] we have that $\Gamma \sim 1/T_2^*$.

Off-resonant case

In the off-resonant case, the magnetic field is applied in the direction X, forming an angle $\theta \approx 55^\circ$ with respect to the direction parallel to the CNT at rest. To model these measurements, we use Eq. (12) with H_0 expanded to first order in χ, see Eq. (5). The simulation of Fig. 2**C** of the main text is done by time evolving the master equation (12) until steady-state is reached and time-averaging the unblocked state probability. For the simulation, the parameters taken are

$$\lambda_1^{(2)} = 433\,\frac{\text{neV}}{\text{nm}}, \quad \bar{\beta} = 2\times10^{-12}, \quad \Gamma = 392.9\,\text{MHz}, \quad \Omega = 2\pi\times7.6\,\text{MHz}. \tag{13}$$

The spin-mechanics coupling $\lambda_1^{(2)}$ is chosen to match the observed EDSR broadening observed at the mechanical resonance. The value of $\bar{\beta}$ is set to match the observed asymmetry in the frequency response of the mechanical oscillator, while the values of Ω and Γ are chosen to match the EDSR width. It is worth noting that within the regime of strong decoherence, $\Gamma \gg \Omega$, particularly precise values Ω and Γ are not too relevant. For the calculations corresponding to the simulation displayed in Fig. 2**C** in the main text only the width of the resonance depends on their value.

Resonant, parallel field case

In the resonant case, the magnetic field is oriented parallel to the CNT at rest. That is, $B_\perp = 0$, $B_{||} = B$. Substituting into Eq.(6) we see that in this case $\lambda_1 = 0$, i.e. the linear contribution of the mechanics vanishes. Therefore, the lowest order contribution from the mechanics is the quadratic term, and the second coupling takes the simple form given in Eq. (3) of the main text.

As described in the main text, looking at the effective frequency shift produced by the mechanics in this case, we extract the analytical expression for the frequency shift given in Eq. (4) of the main text. We fix the values of the resonance $\omega_m/2\pi = 261.9$ MHz, and quality factor to $Q = 5000$ and fit the remaining parameters to the observed frequency shifts. We obtain

$$\bar{\beta} = 2 \times 10^{-11}, \qquad \lambda_2^{(1)}\chi_{\max}^2 = 40.3 \pm 0.8\ \text{MHz}, \qquad \lambda_2^{(2)}\chi_{\max}^2 = 23.1 \pm 0.9\ \text{MHz}, \tag{14}$$

where $\chi_{\max}$ is the maximum displacement of the CNT, the superscript indicates each of the two resonances observed in Fig. 2**C** of the main text, and the values of $g_{||}$ are reported in the main text. It is worth noting that the value of the Duffing coefficient in this case is not exactly the same as the off-resonant one. This is not surprising as previous works have shown that the Duffing non-linearity in a CNT depends strongly on the applied gate voltages.[2,3]

From the parameters of the CNT and the mechanical driving power $P_{\text{d,osci}}$ we estimate a maximum displacement of $1.5\,\text{nm}$, following the same procedure outlined in the Supplementary Material of Ref. [1] (with the same parameters). This gives us the estimate of the second-order coupling constants

$$\lambda_2^{(1)} = 76.5\,\frac{\text{neV}}{\text{nm}^2}, \qquad \lambda_2^{(2)} = 54.3\,\frac{\text{neV}}{\text{nm}^2}. \tag{15}$$

In terms of the zero-point motion, the bare second-order coupling $\kappa_2^{(k)} = \lambda_2^{(k)}\chi_{\text{zpm}}^2$ is

$$\kappa_2^{(1)} = 2\pi \times 8.5\,\text{Hz}, \qquad \kappa_2^{(2)} = 2\pi \times 6.0\,\text{Hz}. \tag{16}$$

References

[1] F. Vigneau, J. Monsel, J. Tabanera, K. Aggarwal, L. Bresque, F. Fedele, F. Cerisola, G.A.D. Briggs, J. Anders, J.M.R. Parrondo, A. Auffèves, and N. Ares, Ultrastrong coupling between electron tunneling and mechanical motion, *Phys. Rev. Res.* **4**, 043168 (2022).

[2] G.A. Steele, A.K. Hüttel, B. Witkamp, M. Poot, H.B. Meerwaldt, L.P. Kouwenhoven, and H.S. van der Zant, Strong coupling between single-electron tunneling and nanomechanical motion, *Science* **325**, 5944, (2009).

[3] A. Castellanos-Gomez, H.B. Meerwaldt, W.J. Venstra, H.S. J. van der Zant, and A.G. Steele, Strong and tunable mode coupling in carbon nanotube resonators, *Phys. Rev. B* **86**, 041402 (2012).

[4] F. Pistolesi, A.N. Cleland, and A. Bachtold, Proposal for a Nanomechanical Qubit, *Phys. Rev. X* **11**, 031027 (2021).

[5] N. Ares, V.N. Golovach, G. Katsaros, M. Stoffel, F. Fournel, L.I. Glazman, O.G. Schmidt, and S. De Franceschi, Nature of Tunable Hole g Factors in Quantum Dots, *Phys. Rev. Lett.* **110**, 046602 (2013).

[6] D. Jirovec, P.M. Mutter, A. Hofmann, A. Crippa, M. Rychetsky, D.L. Craig, J. Kukucka, F. Martins, A. Ballabio, N. Ares, D. Chrastina, G. Isella, G. Burkard, and G. Katsaros, Dynamics of Hole Singlet-Triplet Qubits with Large g-Factor Differences, *Phys. Rev. Lett.* **128**, 126803 (2022).

[7] V.P. Michal, J.C. Abadillo-Uriel, S. Zihlmann, R. Maurand, Y.-M. Niquet, and M. Filippone, Tunable hole spin-photon interaction based on g-matrix modulation, *Phys. Rev. B* **107**, L041303 (2023).

[8] A. Crippa, R. Maurand, L. Bourdet, D. Kotekar-Patil, A. Amisse, X. Jehl, M. Sanquer, R. Laviéville, H. Bohuslavskyi, L. Hutin, S. Barraud, M. Vinet, Y.-M. Niquet, and S. De Franceschi, Electrical Spin Driving by g-Matrix Modulation in Spin-Orbit Qubits, *Phys. Rev. Lett.* **120**, 137702 (2018).

[9] K. Flensberg, and C.M. Marcus, Bends in nanotubes allow electric spin control and coupling, *Phys. Rev. B* **81**, 195418 (2010).

[10] A. Sen, G. Frank, B. Kolok, J. Danon, A. Pályi Classification and magic magnetic field directions for spin-orbit-coupled double quantum dots, *Phys. Rev. B* **108**, 245406 (2023).

[11] T. Pei, A. Palyi, M. Mergenthaler, N. Ares, A. Mavalankar, J. H. Warner, G.A.D. Briggs, and E.A. Laird, Hyperfine and Spin-Orbit Coupling Effects on Decay of Spin-Valley States in a Carbon Nanotube, *Phys. Rev. Lett.* **118**, 177701 (2017).
